# CI-Träger der ersten Generation

**DOI:** 10.1007/s00106-025-01549-9

**Published:** 2025-03-06

**Authors:** Annette Leonhardt, Stefanie Fiocchetta

**Affiliations:** Abteilung für Präventions-, Inklusions- und Rehabilitationsforschung, Ludwig-Maximilians-Universität, Leopoldstr. 13, 80802 München, Deutschland

**Keywords:** Cochlea-Implantat, Erste Cochlea-Implantat-Versorgungen, Motivation für CI-Versorgung, Erfahrungen vor und nach der Implantation, Hörerfolg, Lebensqualität, Cochlear implant, First cochlear implant restorations, Motivation for CI, Experience before and after implantation, Hearing success, Quality of life

## Abstract

In den 1980er-Jahren wurden die ersten Cochlea-Implantationen durchgeführt. Implantiert wurden Personen, die zumeist viele Jahre ertaubt waren. Sie entschieden sich für eine CI-Versorgung, ohne auf Langzeitstudien oder Erfahrungen anderer Personen zurückgreifen zu können. Was war ihre Motivation für eine CI-Versorgung? Welche Erfahrungen machten sie (z. B. mit dem sozialen Umfeld) vor und nach der Implantation? Wie schätzen sie heute ihre Entscheidung von damals ein? Diesen und weiteren Fragen wurde im Rahmen einer qualitativen Studie nachgegangen. Der Leidensdruck der befragten Personen war so groß, dass sie den ungewissen Ausgang nicht scheuten. Es zeigt sich aber auch, dass es bei den CI-Trägern der 1. Generation (definiert als CI-Versorgung bis 1989/1990) um besonders selbstbewusste Persönlichkeiten mit großer Willensstärke, Leistungsbereitschaft und unerschütterlichem Optimismus handelt.

## Abstract (en)

The first cochlear implantations (CI) were carried out in the 1980s. In most cases the recipients had a long history of deafness. They had opted for CI transplant without recourse to any long-term studies or experience of other CI recipients. What was the motivation for their decision? What was their experience (e.g., social environment) before and after the implantation? How do they perceive their decision at this point in time? These and further questions are addressed within the framework of a qualitative study. Such was the extent of suffering of the precochlear recipients that they went ahead regardless of the outcome. As it emerged from our study, the CI recipients of the first generation (defined as CI provision to 1989/1990) were particularly strong-minded personalities with an extremely strong willpower, willingness and utmost optimism.

## Hinführung

In den 1980er-Jahren kam es zu den ersten Cochlea-Implantat(CI)-Versorgungen. Versorgt wurden damals zunächst Menschen mit spät eingetretenem Hörverlust [9], die – zumeist nach progredienter Schwerhörigkeit – ertaubt waren und schon über viele Jahre nicht mehr oder nur unter erheblicher (Hör‑)Anstrengung und unter Zuhilfenahme des Absehens am kommunikativen Geschehen der sie umgebenden hörenden Umwelt teilhaben konnten. Zu diesem Zeitpunkt lagen weder Erfahrungen noch wissenschaftliche Studien über den Erfolg und die langfristigen Auswirkungen von Implantationen vor. Daher ist es von Interesse, der Frage nachzugehen, warum sich diese Personen dennoch mit CI versorgen ließen.

## Hintergrund

Einbezogen wurden Menschen, die prälingual und erst im späten Jugend- oder im Erwachsenenalter schwerhörig wurden. Nicht selten handelt es sich um progredient verlaufende Schwerhörigkeiten, die in einer Ertaubung münden. Diese Menschen befinden sich in mehrerlei Hinsicht in einer besonderen Situation. Sie erleben

- einen Verlust der bisher für sie als „normal“ geltenden und als gesichert empfundenen Lebenswelt,
- sich in neuen, veränderten Kommunikationssituationen, die sie als (schwerwiegende) Kommunikationsbehinderung wahrnehmen und
- nicht selten den Verlust des sozialen Status.

Richtberg [15] wies nach, dass es bei diesem Personenkreis gehäuft zu psychovegetativen Störungen kommt. Erschwerend wirkt die Unsichtbarkeit der Hörbeeinträchtigung, die dazu führt, dass Verständnisschwierigkeiten vom Umfeld als „Begriffsstutzigkeit“ eingeordnet werden und andere (nicht sofort zu erkennende oder plausible) Verhaltensweisen realisiert werden, aber diese nicht erklärbar sind. Nicht selten vermeiden Menschen mit spät eingetretenem Hörverlust, ihre Bedürfnisse in der Öffentlichkeit mitzuteilen sowie Hör- und/oder Kommunikationstaktik anzuwenden. Ebenso versuchen sie oft, ihre Hörbehinderung zu vertuschen und teilweise auch zu verdrängen. Dieses Verhalten führt zu weiteren Missverständnissen und bringt nicht selten Fehlbeurteilungen mit sich.

Zu Beginn der 1980er-Jahre tauchten die ersten Cochlea-Implantate, die weitestgehend den heutigen Modellen im Aufbau und in der Funktionsweise entsprachen, auf dem Markt auf. Deutliche Unterschiede gab es noch im Tragekomfort. Der Sprachprozessor (in Größe einer Zigarettenschachtel) musste in der Hemd- oder Blusentasche oder am Gürtel getragen werden. Für den Träger wirkte das erforderliche Verbindungskabel nicht selten störend bzw. beeinträchtigend.

Mit einer Implantation verbunden ist (neben dem üblichen Operationsrisiko) eine Operation „am Kopf“, wie es die Interviewpartner formulierten, was für sie zu einer (zusätzlichen) Verunsicherung führte. Die ersten Personen, die sich mit einem Cochlea-Implantat versorgen ließen, widerstanden nicht nur dieser Verunsicherung, sondern sie konnten auch nicht auf persönliche Erfahrungen anderer oder auf Ergebnisse aus Langzeitstudien zurückgreifen. Ebenso konnten Fragen nach den Risiken der Implantation, der möglichen Liegedauer des Implantats, der Ausfallquote des Implantats, der Möglichkeit einer Reimplantation und Wiederversorgung sowie nach den Erfolgsaussichten auf ein offenes Sprachverstehen letztendlich nicht sicher beantwortet werden. Trotz des Fehlens von Antworten auf möglicherweise bestehende Fragen ließen sich erste Personen implantieren. Dieser Personenkreis wird in der vorliegenden Studie als „CI-Träger der 1. Generation“ definiert. Der Implantionszeitraum umfasst für diese Gruppe den Zeitpunkt von Beginn der ersten Implantat-Versorgungen bis 1989/90.

## Ziel der Arbeit/Fragestellungen

Um über diesen Personenkreis und deren Motivation zu diesem Schritt mehr zu erfahren, wurde im Konkreten drei Fragestellungen nachgegangen:

1. Was war die Motivation für die CI-Versorgung?
2. Welche Erfahrungen machten sie (z. B. mit dem sozialen Umfeld) vor und nach der Implantation? Wie reagierte das Umfeld?
3. Wie schätzen die CI-Träger der 1. Generation heute ihre Entscheidung von damals ein?

## Untersuchungsteilnehmer und Methode

Die Daten wurden durch Fallstudien im Rahmen von Forschungsarbeiten (Bachelor-Arbeiten) in den Studiengängen „Prävention, Inklusion und Rehabilitation (PIR) bei Hörschädigung (B. Sc.)“ und „Prävention, Inklusion und Rehabilitation (PIR) bei Hörschädigung (Modellstudiengang) (B. Sc.)“ am Lehrstuhl für Gehörlosen- und Schwerhörigenpädagogik der LMU München erhoben. Dazu wurden mit den Teilnehmern leitfadengestützte Einzelinterviews durchgeführt; diese wurden zunächst je Fall und nachfolgend übergreifend systematisiert und analysiert (11 davon durch eine komparative Studie im Rahmen einer Master-Arbeit [11]). Es handelt sich um eine rein qualitative Studie.

Befragt werden konnten (bis 2022) 14 Personen, die sich im Zeitraum bis 1990 mit einem Cochlea-Implantat versorgen ließen. Begonnen wurde mit den ersten Befragungen im Jahr 2017. Mit einer Erweiterung der Untersuchungsgruppe ist nicht mehr zu rechnen, da einerseits seit Beginn der Erhebung intensiv nach potenziellen Teilnehmern gesucht wurde und andererseits ein Teil der CI-Träger der 1. Generation aufgrund der Altersstruktur nicht mehr für ein Interview zur Verfügung steht.

Die befragten Personen entstammten den Geburtsjahrgängen 1939–1950. Das genaue Geburtsdatum wurde aus Datenschutzgründen nicht erfasst. Die meisten Interviewpartner nannten dennoch ihr Geburtsjahr; bei anderen konnte es aufgrund weiterer Angaben „in etwa“ ermittelt werden. Sie waren zum Zeitpunkt der Erstversorgung ca. 30 bis Mitte 40 Jahre alt. Zum Interviewzeitpunkt waren sie zwischen 66 und 78 Jahre alt. Teilgenommen haben 9 Frauen und 5 Männer.

Die Ertaubungsdauer lag zwischen 6 Monaten und 34 Jahren. Zwei weitere Personen waren seit 40 Jahren ertaubt, wovon eine bis zur Versorgung über ein Restgehör verfügte und die andere mit 2 Jahren ertaubt war und daher nicht zur eigentlichen Zielgruppe gehörte. Ohne diese beiden Personen war die untersuchte Gruppe durchschnittlich 14 Jahre taub, bevor es zur CI-Versorgung kam. (Konkret waren je eine Person 6 Monate, ein Jahr sowie 2, 7, 16, 18, 19, 23, 27 und 34 Jahre sowie je 2 Personen 11 Jahre ertaubt. Hinzu kommen die beiden Personen mit 40 Jahren.) Die Implantationsorte waren Wien (1980, 1982 und 1984), Düren 1984 (wobei es hier zu einer zeitnahen „Neuversorgung“ in Hannover kam), Berlin (Charité) (DDR) 1985 und 1988, München 1988 und Hannover 1984, 1985, 1986 (2‑mal), 1988, 1989, Tab. 1. Ein Befragungsteilnehmer nannte sein Implantationsjahr nicht; durch die Vorerhebung war jedoch sichergestellt, dass die Person der Zielgruppe angehörte.

**Tab. 1:** Implantationsjahre und -orte

Implantationsjahr	Implantationsort
1980	Wien
1982	Wien
1984	Wien, Düren, Hannover, einmal o. A.
1985	Hannover, Berlin (Charité)
1986	Hannover (2-mal)
1988	Hannover, München, Berlin (Charité)
1989	Hannover

## Ergebnisse und Diskussion

Ausgewertet wurde nach Erfahrungen vor und nach der Implantation, der Motivation für die CI-Versorgung und der Einschätzung der (damaligen) Entscheidung aus heutiger Sicht.

### Erfahrungen vor der Implantation

Die Erfahrungen vor der Implantation wurden nach den Aspekten Hörverlust, schulischer und beruflicher Werdegang, Kommunikation, soziales Umfeld, Alltags- und Freizeitgestaltung, psychosoziale Auswirkungen, Kontakte zu anderen Menschen sowie Bewältigung der Situation strukturiert und ausgewertet.

#### Hörverlust

Bei den CI-Trägern der 1. Generation handelt es sich um Personen mit frühzeitig eingetretener Schwerhörigkeit (nach Meningitis, Scharlach, Mumps) mit progredientem Verlauf und um Personen mit (rascher) Ertaubung nach Unfall und Virusinfektion.

#### Schulischer und beruflicher Werdegang

Im persönlichen Werdegang der Interviewten zeigt sich ein breites Spektrum von „kein Einfluss der Hörschädigung“ auf den schulischen Werdegang über Schulwechsel oder Abbruch des Studiums bis hin zur Arbeitsunfähigkeit. Unabhängig vom schulischen Werdegang stand ihnen nur eine eingeschränkte Auswahl an Berufen zur Verfügung. Bei deren Ausübung erlebten sie weitere Einschränkungen, wobei das „Nicht-telefonieren-Können“ am häufigsten genannt wurde. Bei der Mehrzahl zeigte sich, spätestens ab Eintreten eines bestimmten Ausmaßes des Hörverlustes, ein „Knick in der Lebenslinie“.

#### Kommunikation

Die Kommunikation erfolgte über das Absehen (teilweise auch unbewusst) und ausschließlich lautsprachlich. Ihnen war bewusst, dass es aufgrund der fehlenden auditiven Eigenkontrolle zu Veränderungen bei den eigenen lautsprachlichen Äußerungen kam (genannt wurde v. a. zu lautes Sprechen). Ein Teilnehmer des Interviews unternahm den Versuch, die Gebärdensprache zu erlernen; ein weiterer benutzte bei der Kommunikation neben der Lautsprache das Fingeralphabet.

#### Soziales Umfeld

Auffallend war, dass alle interviewten CI-Träger gut in ihre Familien eingebettet waren. Zu Problemen kam es mitunter mit ehemaligen Freunden und Personen „von außerhalb“. Sie fühlten sich von ihnen nicht ausreichend verstanden. Missverständnisse in der Kommunikation führten dazu, dass man sich von ihnen zurückzog.

#### Alltags- und Freizeitgestaltung

Die Befragten gaben an, dass sie bei ihrer Alltags- und Freizeitgestaltung eingeschränkt waren; so wurde beispielsweise genannt, die Pointen beim Fernsehen oder bei Veranstaltungen verpasst zu haben. Erhebliche Einschränkungen empfanden sie bei musikalischen Aktivitäten, an denen sie dann auch nicht mehr teilnahmen.

#### Psychosoziale Auswirkungen

Der Eintritt der Ertaubung und das nachfolgend eingetretene Bewusstsein, dass das Hören unwiederbringlich „verloren“ ist, wurden als „Schock“ und „Trauma“ beschrieben. Trotz des erwähnten guten familiären Anschlusses fühlte man sich aus dem sozialen Umfeld, dem man bis dahin angehörte, ausgeschlossen, mit der Folge, dass man sich noch mehr auf die eigene Familie konzentrierte. Allerdings zeigte sich gerade in diesem Bereich ein ausgesprochen individuelles Erleben der Situation.

#### Kontakte mit hörenden, schwerhörigen und ertaubten Menschen

Trotz der zunehmenden Schwerhörigkeit und der dann eintretenden Ertaubung bestanden Kontakte v. a. zu Hörenden, nur selten zu Schwerhörigen und Ertaubten und nur im Einzelfall zu Menschen mit Gehörlosigkeit/​Taubheit. Ihre Sozialisation hatten sie in der Welt der Hörenden erfahren; sie verblieben in dieser, auch wenn sie an dieser nur noch eingeschränkt teilhaben konnten.

#### Bewältigung der Situation

Die Situation wurde in erster Linie dadurch bewältigt, dass die Befragten die Hoffnung auf Heilung bzw. Verbesserung ihrer (Hör‑)Situation nicht aufgaben. Nicht wenige suchten wiederholt verschiedene Ärzte auf, immer verbunden mit der Erwartung und Hoffnung, dass diese doch eine neue und weiterführende Information hätten.

Zusammenfassend kann festgehalten werden, dass die Befragten große Probleme in kommunikativer Hinsicht hatten und eine Verständigung kaum möglich war. Die letztendlich irgendwann eingetretene Ertaubung mit dem Bewusstsein der Endgültigkeit des vollständigen Hörverlustes wurde als „ein ganz schweres Trauma“ und „seelischer Schock“ beschrieben. Durchgängig wurde eine Abhängigkeit von den Ehepartnern und Kindern genannt.

### Motivation

Da das CI zu dieser Zeit noch völlig unbekannt war, interessierte zunächst die Frage, wie bzw. wodurch die Befragten vom Cochlea-Implantat erfahren haben. Etwa die Hälfte (konkret 7) gaben an, davon aus Printmedien erfahren zu haben, 2 nannten das Fernsehen und 6 „von anderen Privatpersonen“. Zwei erhielten die Information von dem behandelnden Arzt (Tab. 2).

**Tab. 2:** Woher erfuhren die Interviewpartner vom Cochlea-Implantat? (Ein Befragter gab 2 Quellen an)

Informationsquelle	Anzahl
Aus den Printmedien	7
Aus dem Fernsehen	2
„Von anderen Personen“	6

3 der interviewten CI-Träger konnten sich mit (bereits) mit dem Implantat versorgten Personen vor der Operation austauschen, jedoch erst unmittelbar vor der eigenen Versorgung, sodass dies auf die Entscheidungsfindung keinen Einfluss mehr nahm.

Mehrere Befragte gaben an, dass sie vom Cochlea-Implantat erfuhren und dann direkt zu einer der (damals sehr wenigen die CI‐Versorgung durchführenden) Kliniken fuhren, um sich vor Ort zu informieren.

Die Situation (vor der CI-Versorgung) wurde von allen als „unerträglich“ beschrieben, was sich auch in den persönlichen Aussagen widerspiegelt: „Ich … hatte nichts zu verlieren“ [1, 5, 12, 13], „… es konnte prinzipiell nur besser werden“ [10], „Ich höre so nicht und wenn’s nichts wird, dann hörnwa auch nicht“ [2], „Mehr wie taub kann ja nicht werden“ [4], „Noch schlimmer als taub sein geht nicht“ [14]. „… wieder hören [können], stand an erster Stelle“ [4], „… konnte nur gewinnen“ [1, 5] oder „Da muss es doch was geben, dass es besser wird“ [16]. Die Verzweiflung und umfassende Motivation, „um jeden Preis“ wieder „hören zu können“, zeigt sich auch in folgenden Aussagen: „Hauptsache wieder … hören und ALLES (Schreibung in Großbuchstaben, da im Interview besonders betont/hervorgehoben), was da geht“ [16], „Ich habe VERZWEIFELT nach Hilfe gesucht. Ich habe den Bekannten gesagt, wenn ihr IRGENDETWAS hört, was man machen kann, bitte melden“ [7] und „Eigentlich hatte ich gar keine feste Vorstellung, was nachher kam, ich wollte nur wieder etwas hören“, weshalb die befragte Person, nachdem sie in der Zeitung vom CI gelesen hatte, „sofort nach dem rettenden Strohhalm gegriffen“ [4] hat. Nachrangig kam von einigen auch das Interesse am technischen und am wissenschaftlichen Fortschritt dazu.

Wertet man *zusammenfassend*, war die häufigste Aussage „ich konnte nur gewinnen, nichts verlieren …“, gefolgt von „wieder hören können“ und „wieder kommunizieren können“. Letztendlich war die CI-Versorgung der selbstgewählte „Sprung ins kalte Wasser“ [14] oder „… ein Sprung ins grüne Nass“ [1]. Die Leidensdruck war so groß, dass auch der Rat anderer HNO-Ärzte, die das damals noch weitestgehend unbekannte CI nicht kannten und vehement davon abrieten, nicht befolgt wurde: „Ja, der … hat viel später noch Papiere verfertigt, …, dass das alles nur mehr eine Einbildung wäre, aber eigentlich nichts Richtiges …“ [16].

### Erfahrungen nach der Implantation

Auch hier erfolgt wieder eine Untergliederung entsprechend den gewählten Auswertungsbereichen, die sich auf die Erstanpassung (des Sprachprozessors), das Hörtraining und die Reha, das Hören mit CI, die Kommunikation, die Auswirkungen auf die berufliche Tätigkeit, die Reaktionen des sozialen Umfelds, die Alltags- und Freizeitgestaltung, die psychosozialen Auswirkungen sowie den Kontakt zu anderen CI-Trägern beziehen.

#### Erstanpassung

Die Erstanpassung war zumeist mit einem positiven und emotionalen Gefühl verbunden. Sie wurde mit „beglückend“ [1], „fantastisch“ [6], „überwältigend“ [4, 10], „euphorisch“ [8], „mir standen … die Tränen in den Augen“ [12] und „war sprachlos“ [13] beschrieben. Beeindruckt waren die Befragten auch vom Wahrnehmen der eigenen Stimme „… ich habe meine Stimme gehört und konnte die Stimme von … und … und … unterscheiden“ [10] sowie „Wahnsinn! … ich hab mich selbst gehört und hab mich nicht erkannt, weil ich viel zu laut war“ [4].

#### Hörtraining und Reha

Ganz besonders in diesem Bereich zeigte sich, dass die CI-Versorgung zu diesem Zeitpunkt noch in den Anfängen steckte. Teilweise fand kein Hörtraining bzw. keine Reha statt, oder die Probanden konnten sich an ein solches nicht erinnern. Wenn ein solches stattfand, entsprach es nicht heutigen Standards und Vorstellungen. Allerdings haben viele von ihnen privat zu Hause geübt, um Fortschritte im Hören zu erreichen.

#### Hören mit CI

Konsens war, dass das Hören mit CI nach jeder Anpassung besser wurde. Im Einzelfall kam es zum sofortigen Sprachverstehen nach der Erstanpassung. Die CI-Träger gaben an, dass sie zunehmend deutlich weniger Schwierigkeiten bei der Kommunikation hatten. Als Hörfortschritte wurde das Hören können der eigenen Schritte erwähnt; teilweise waren auch Fernsehen und Telefonieren wieder möglich.

#### Kommunikation

Bei der Umsetzung der Kommunikation zeigten sich sehr individuelle Erfahrungen und Unterschiede; gemeinsames Merkmal war jedoch, dass sie das Cochlea-Implantat als Erleichterung für ihre Lebensgestaltung und hilfreich für die Kommunikation empfanden. Es wurde ein Sprachverstehen möglich, wobei bei Mehreren der Höreindruck durch das Absehen ergänzt wurde.

#### Berufliche Tätigkeit

Mehrere der Befragten konnten eine durch erweiterte Aufgaben gekennzeichnete neue Position – v. a. durch die nun möglichen Kundenkontakte – erringen. Selbstständiges Telefonieren wurde möglich. Für einzelne tat sich ein völlig neues Tätigkeitsfeld auf, so z. B. im Rahmen der neu gegründeten Deutschen Cochlea Implant Gesellschaft (DCIG) (heute Deutsche Cochlea Implantat Gesellschaft).

#### Soziales Umfeld

Wie unter „Erfahrungen vor der Implantation“ ausgeführt, waren alle Befragten gut in ihre Familien eingebettet. Dieses positive Familienklima hat sie bei der Entscheidungsfindung ein Stück weit getragen und trug zweifelsohne zu einer positiven Entwicklung nach der CI-Versorgung bei. Bei den Befragten kam es zu einer Erweiterung des Freundes- und Bekanntenkreises. Das CI wurde als Gewinn und als so bereichernd empfunden, dass der Operationstermin nachfolgend als „Geburtstag“ [4] gefeiert wird.

In einem Fall zerbrach die Ehe. Das Konstrukt, auf dem sie bestand, war durch die wachsende Selbstständigkeit des implantierten Ehepartners offensichtlich nicht mehr gegeben.

#### Alltags- und Freizeitgestaltung

Ein Teil der CI-Träger betonte, dass Musik hören (wieder) eine größere Rolle spielte; andere erwähnten, dass gerade das Musik hören ein Bereich ist, den sie leider nicht wieder genießen können, und sahen das auch als einen echten Verlust an.

Ein Teil der CI-Träger engagierte sich ehrenamtlich, insbesondere in der Selbsthilfe oder in der DCIG und/oder in einem ihrer Regionalverbände.

#### Psychosoziale Auswirkungen

Aus den Interviews wurde deutlich, dass die befragten Teilnehmer nach der CI-Versorgung selbstsicherer, selbstständiger und kontaktfreudiger wurden und sich als Persönlichkeit weiterentwickelten. Sie äußerten sich insgesamt zuversichtlich, und es war eine sichtliche Lebensfreude wahrnehmbar.

#### Kontakt zu anderen CI-Trägern

Auffällig ist bei diesem Personenkreis, dass sich nahezu alle intensiv in der Selbsthilfe und in neu gegründeten Vereinen (im Bereich der CI-Rehabilitation) engagierten. Zwischen ihnen und den ihnen unmittelbar nachfolgenden CI-Trägern, den CI-Trägern der 2. Generation (von uns definiert für den Implantationszeitraum 1990–1995) besteht ein enges und intensives Netzwerk.

#### Weitere Aussagen

Zum Zeitpunkt der Interviews trug die Mehrheit der befragten Personen nach einer Reimplantation durch Ausfall inzwischen neue Implantate und ist heute bilateral versorgt. Drei der Interviewten trugen noch das Erstimplantat.

Betrachtet man die Erfahrungen nach der Implantation *zusammenfassend*, so kann festgehalten werden, dass das erste Hörerlebnis „einfach unglaublich“ [10] war. Nach viel Übung und mit umfassender Eigeninitiative kam es bei den Personen zu einem zunehmend besseren Sprachverstehen. Ebenso wird erwähnt, dass sich die eigene Sprachproduktion (insbesondere in Hinsicht auf die eigene Sprechlautstärke) verbesserte.

Es kann eingeschätzt werden, dass die neuen beruflichen Perspektiven, die sich auftaten, weniger auf der CI-Versorgung „an sich“ als vielmehr aus dem neu geschöpften Mut, dem gewonnenen Selbstbewusstsein und einer sich erweiternden Selbstständigkeit ergaben.

Sehr unterschiedlich fielen die Aussagen zum Musikhören und zum Telefonieren aus. Während ein Teil dieses (wieder‑)​erlangte und das als äußerst bereichernd und beglückend empfand, haben andere das nicht erreichen können und erleben das als eine (wenn auch akzeptable) Einschränkung.

### Einschätzung der Entscheidung

13 der 14 Befragten nutzen ihre CI und hielten ihre Entscheidung für richtig. Eine befragte Person ist Non-User. Es handelt sich hier um die Person, die im 2. Lebensjahr an einer Meningitis ertaubt war und daher (wie eingangs erwähnt) – aufgrund ihrer prälingualen Ertaubung – eigentlich nicht den festgelegten Untersuchungskriterien entsprach. Dennoch hielt auch sie ihre Entscheidung für richtig, obwohl sie mit dem CI keine Hörerfolge verbuchen konnte: „Ich bereue es nicht. Ich würde es jedem empfehlen. … Die Entscheidung liegt immer bei einem selbst“ [5].

An der Richtigkeit der Entscheidung wurde von keiner Person gezweifelt (auch nicht trotz Reimplantationen und Komplikationen sowie inzwischen veralteten Implantaten mit weniger Tragekomfort, eingeschränkter Leistungsfähigkeit und in Einzelfällen mit nur einseitiger Versorgung).

Die nachfolgenden Zitate belegen, dass sich die Erwartungen für die Befragten erfüllt haben und sie zugleich eine sachliche und realistische Haltung zum Cochlea-Implantat haben: „… das CI hat mir viel gebracht, wenn auch nicht alles …“ [8], „… alleine für die Wahrnehmung der Geräusche gelohnt“ (nach [4]), „… ich kann ohne das Ding nicht mehr sein. … Wenn das morgen kaputtgeht, dann fahr ich wieder nach … und will ein neues haben. … Es ist UNVORSTELLBAR“ [4] und „War das Beste, was mir je passieren konnte“ [6] oder „Zu 150 % ist es ein Segen. Etwas Besseres gibt es nicht“ [14]. Selbst der Non-User wertet seinen Versuch, mit dem CI zu einem Hören zu kommen, positiv: „Aber ich bereue es nicht. Ich würd’ es jedem empfehlen“ [5].

Aber auch bei den erfolgreichen Nutzern des CI blieben Wünsche offen, oder es haben sich nicht alle Erwartungen erfüllt: „Ich möchte gern Operetten hören. … Operetten hören ist wie Katzen-Gejaule“ [3] und „Das ist Musik hören. Das geht nicht“ [8] oder „… telefonieren konnte ich nie … Auch jetzt nicht, das quäkt mir zu sehr“ [1].

#### Abschließende Bemerkungen

Bei dem Personenkreis der CI-Träger der 1. Generation handelte es sich um hoch motivierte und hoch interessierte Personen, die dem Status des Nicht-hören-Könnens um nahezu jeden Preis „entfliehen“ wollten. Sie sehen sich selber als „Pioniere der CI-Versorgung“ und haben sich nach der Implantation intensiv engagiert, um das Cochlea-Implantat bekannt(er) werden zu lassen. Dazu gehörten u. a. das Entstehen von Selbsthilfegruppen, die Gründung der DCIG e. V. und die Herausgabe der Zeitschrift *Schnecke*. Zwischen vielen CI-Trägern der Anfangszeit (CI-Träger der 1. und 2. Generation) besteht eine enge Verbundenheit. Es handelte sich zumeist um ungewöhnlich taffe, selbstbewusste Persönlichkeiten mit großer Willensstärke, Leistungsbereitschaft und unerschütterlichem Optimismus, die zuvor auch ihre hochgradige Hörschädigung bzw. Ertaubung gut gemeistert hatten, beruflich trotz Hörbehinderung erfolgreich waren und Problemen sowie Unwegsamkeiten nicht aus dem Weg gingen, sondern sich aktiv mit diesen auseinandersetzten. Beispielsweise legten einzelne trotz zunehmender Schwerhörigkeit das Abitur ab und begannen ein Studium. Andere gingen bewusst allein zum Einkauf, obwohl sie wussten, dass sie mit ihrer Sprechweise beim Metzger „auffielen“. Sie hatten sich nach ihrer Ertaubung Strategien angeeignet, mit der sie unterschiedliche Kommunikationssituationen meistern konnten. Sie handelten selbstbewusst und eigenständig und übertrugen das auch auf die CI-Versorgung. Selbst bei ablehnender Haltung des Ehepartners und der Kinder [10] ließen sie sich vom geplanten Vorhaben nicht abbringen. Aus der CI-Versorgung und dem eintretendem Hörerfolg schöpften sie weiteres Vertrauen in ihre Leistungsfähigkeit und nahmen z. B. ein abgebrochenes Studium wieder auf und promovierten.

Ein wesentlicher Teil des Erfolgs bestand offensichtlich in der geringen Erwartungshaltung: „… ich habe auf keinen Fall zu große Erwartungen gehabt“ [14], „Ein besseres Sprachverständnis, ein besseres Ablesen vom Mund, das [habe] ich mir erhofft. Ich konnte selbst überhaupt nicht richtig glauben, dass ich jemals wieder hören können würde“ [8] oder „Es war eigentlich ein tolles Erlebnis, ÜBERHAUPT wieder was zu hören“ [7]. Gleiches bewirkte der eher sachliche Umgang der Mediziner über den zu erwartenden Hörerfolg: „… kann nicht versprechen, dass [Sie] mit diesem Hören zufrieden sind“ [10], „Die haben dir damals gesagt, du würdest was hören können, aber … nicht ganz sicher, ob du gutes Sprachverstehen hast“ [6], „Die Ärzte haben keineswegs gesagt, dass ich Sprache verstehen würde“ [16] oder „Man KANN mir nicht sagen, wie das Ergebnis ausfallen [wird]“ [7]. Im Gegensatz zu heute waren viele der Befragten seit Langem ertaubt, wodurch der ehemalige Höreindruck verblasst war. Sie wollten ihre Lebenssituation (insbesondere die Kommunikationssituation) aktiv verändern. Eine Möglichkeit sahen sie in dem Versuch, sich mit einem Cochlea-Implantat versorgen zu lassen. Das Fehlen von Langzeitstudien empfanden sie nicht als Hinderungsgrund. Da sie mit gemäßigten Erwartungen an die CI-Versorgung herangingen, war die Freude über die (langfristig eingetretenen) Hörerfolge besonders groß.

## Fazit

- Die CI-Träger der 1. Generation profitierten von der vorab geführten Aufklärung, dass das CI kein „Allheilmittel“ ist und der Erfolg des Hörens nicht vorhergesagt werden kann. Die Bedeutung und Tragweite des nach der Versorgung notwendigen Hörlern- bzw. Hör„um“lernprozesses sollte daher auch heute in Beratungsgesprächen umfassend thematisiert werden.
- Die Phase der hochgradigen Schwerhörigkeit und des Taubseins (Zeitpunkt des Hörverlustes bis zur CI-Versorgung) ist heute deutlich kürzer, v. a. nach plötzlicher Ertaubung durch Krankheit oder Unfall. Das akustische Erinnerungsvermögen ist bei diesem Personenkreis weniger verblasst, sodass der gewohnte Höreindruck noch präsenter ist und so auch „erwartet“ wird. Mit dem Implantat bekommt der Patient ein (technisches/künstliches) Hörvermögen, was jedoch vom Vorherigen abweicht.
- Das persönliche Engagement und die Willensstärke des Einzelnen (im Sinne einer Eigenverantwortung für sich selbst) tragen zum Erfolg bei. Dies gilt selbstverständlich auch – bei entsprechender Beratung und Begleitung – für Menschen mit Migrationsbiografie. Ebenso können Senioren mit (altersbedingter) hochgradiger Schwerhörigkeit und Personen mit Restgehör Nutznießer einer CI-Versorgung sein, zumal ihnen lange Zeit Höreindrücke zur Verfügung standen.
- Die Erfahrungen der CI-Träger der 1. Generation bieten Impulse für alle Personengruppen mit hochgradiger Höreinschränkung.

## Data Availability

Die in dieser Studie erhobenen Datensätze können auf begründete Anfrage beim Korrespondenzautor angefordert werden.

## References

[1] Barta G (2018a) CI-Träger der „1. Generation“. Eine Einzelfallstudie. Bachelorarbeit zur Erlangung des B. Sc. Prävention, Inklusion und Rehabilitation (PIR) bei Hörschädigung (unveröff.), München

[2] Barta G (2018b) CI-Träger der 1. Generation. Unveröfftl. Skript

[3] Fischer B (2017) CI-Träger der 1. Generation. Eine Fallstudie. Bachelorarbeit zur Erlangung des B. Sc. Prävention, Inklusion und Rehabilitation (PIR) bei Hörschädigung (Modellstudiengang) (unveröff.), München

[4] Götzensberger L (2018) CI-Träger der ersten Generation. Eine Einzelfallstudie. Bachelorarbeit zur Erlangung des B. Sc. Prävention, Inklusion und Rehabilitation (PIR) bei Hörschädigung (unveröff.), München

[5] Grund J (2017) Cochlea-Implantat-Träger der ersten Generation. Eine Einzelfallstudie. Bachelorarbeit zur Erlangung des B. Sc. Prävention, Inklusion und Rehabilitation (PIR) bei Hörschädigung (unveröff.), München

[6] Huber S (2018) Cochlea Implantat Träger der ersten Generation. Eine Einzelfallstudie. Bachelorarbeit zur Erlangung des B. Sc. Prävention, Inklusion und Rehabilitation (PIR) bei Hörschädigung (unveröff.), München

[7] Krauß A (2021) CI-Träger der ersten Generation. Eine Einzelfallstudie. Bachelorarbeit zur Erlangung des B. Sc. Prävention, Inklusion und Rehabilitation (PIR) bei Hörschädigung (unveröff.), München

[8] Kutzi M (2018) CI-Träger der ersten Generation. Eine Einzelfallstudie. Bachelorarbeit zur Erlangung des B. Sc. Prävention, Inklusion und Rehabilitation (PIR) bei Hörschädigung (unveröff.), München

[9] Leonhardt A (2023) Menschen mit späterworbener Schwerhörigkeit. In: Leonhardt A, Kaul Th (Hrsg) Grundbegriffe der Hörgeschädigtenpädagogik. Ein Handbuch. Kohlhammer, Stuttgart, S 79–82

[10] Mäck L (2018) CI-Träger der 1. Generation. Eine Einzelfallstudie. Bachelorarbeit zur Erlangung des B. Sc. Prävention, Inklusion und Rehabilitation (PIR) bei Hörschädigung (unveröff.), München

[11] Mäck L (2020) CI-Träger der 1. Generation. Eine vergleichende Fallanalyse. Masterarbeit zur Erlangung des M. Sc. Prävention, Inklusion und Rehabilitation (PIR) bei Hörschädigung (Modellstudiengang) (unveröff.), München

[12] Metz M (2019) CI-Träger der 1. Generation – eine Einzelfallstudie. Bachelorarbeit zur Erlangung des B. Sc. Prävention, Inklusion und Rehabilitation (PIR) bei Hörschädigung (unveröff.), München

[13] Mondry A (2017) CI-Träger der 1. Generation. Eine Fallstudie. Bachelorarbeit zur Erlangung des B. Sc. Prävention, Inklusion und Rehabilitation (PIR) bei Hörschädigung (Modellstudiengang) (unveröff.), München

[14] Radnoti M (2017) CI-Träger der 1. Generation. Bachelorarbeit zur Erlangung des B. Sc. Prävention, Inklusion und Rehabilitation (PIR) bei Hörschädigung (unveröff.), München

[15] Richtberg W (1980) Hörbehinderung als psychosoziales Leiden. Forschungsbericht. Herausgeber: Der Bundesminister für Arbeit und Soziales, Bonn

[16] Schuhmann M (2019) CI-Träger der ersten Generation. Eine Einzelfallstudie. Bachelorarbeit zur Erlangung des B. Sc. Prävention, Inklusion und Rehabilitation (PIR) bei Hörschädigung (unveröff.), München

[17] Weckler J (2017) CI-Träger der Ersten Generation. Eine Fallstudie. Bachelorarbeit zur Erlangung des B. Sc. Prävention, Inklusion und Rehabilitation (PIR) bei Hörschädigung (unveröff.), München

